# Comparison of efficiency and safety between dual-clip and rubber band-assisted ESD and conventional ESD for colonic lateral spreading tumors (LSTs) with different levels of technical difficulty: a retrospective case–control study

**DOI:** 10.1186/s12876-022-02530-4

**Published:** 2022-11-16

**Authors:** Xingbin Ma, Huaiyuan Ma, Tao Gao, Jingrun Cao, Chengxia Liu, Qiong Niu

**Affiliations:** 1 Department of Gastroenterology and Hepatology, Binzhou Medical University Hospital, No. 661 Huanghe 2nd Road, Binzhou, Shandong Province China; 2 Department of Endoscopy Center, Binzhou Medical University Hospital, Binzhou, Shandong Province China

**Keywords:** Endoscopic submucosal dissection, Dual-clip and rubber band-assisted, Comparison, Lateral spreading tumor, Propensity score matching

## Abstract

**Background:**

Dual-clip and rubber band-assisted endoscopic submucosal dissection (DCRB-ESD) is a useful technique in the management of lateral spreading tumors (LSTs) of the colon and is suggested by researchers compared with conventional ESD (C-ESD). The aim of this retrospective study is to further analyze the efficiency and safety of DCRB-ESD in a setting with varying technical difficulties.

**Methods:**

Patients who underwent endoscopic treatment (DCRB-ESD or C-ESD) due to LSTs between Jan 1st, 2019 and Jan 1st, 2022, were retrospectively collected. Patients were classified into the following two groups: the DCRB-ESD group (n = 46) and the C-ESD group (n = 81). Baselines were compared and propensity score matching (PSM) was employed to manage the heterogeneity. The technical difficulty and outcomes of the two groups were evaluated based on a semiquantitative model (CS-CRESD) previously described.

**Results:**

The baseline characteristics of the two groups were balanced except sex and LST classification before PSM and were corrected after PSM. The median ESD operation time of DCRB-ESD was shorter than that of C-ESD (32 vs 41 and 30 vs 44 before and after PSM respectively, P < 0.05). The operation durations of cases with different CS-CRESD scores were different (P < 0.05). In the subgroup with a score of 0, DCRB-ESD showed no advantage than C-ESD in terms of operation duration before and after PSM. In subgroups with a score of 1–3, DCRB-ESD was faster than C-ESD. In subgroups with a score of 4–5, the between-group operation duration was not significantly different due to the limited number of cases, although the median time of DCRB-ESD was shorter. The R0 resection rates, curative resection, complications, and additional surgery in both groups were not significantly different. No adverse events, such as a clip falling off or rubber band rupturing occurred during this study.

**Conclusion:**

DCRB-ESD was an efficient and safe procedure in the management of colonic LSTs. With DCRB-ESD, the operation duration of difficult cases can be shortened without sacrificing complication risk. However, not all cases would benefit from DCRB-ESD. For easy cases (CS-CRESD score = 0), DCRB-ESD may not be prior to C-ESD by experienced endoscopists. A pre-ESD technical difficulty evaluation was recommended to decide whether to perform DCRB-ESD or not.

## Background

Laterally spreading tumors (LSTs) are defined as nonpolypoid lesions spreading laterally rather than vertically with a diameter ≥ 10 mm [1]. Based on the Japan Gastroenterological Endoscopy Society (JSGE) clinical practice guideline published in March 2021 [2], LSTs are classified as granular type (LST-G) and nongranular type (LST-NG) morphologically. Pathologically, LSTs are adenocarcinomas or sessile serrated adenomas/polyps (SSA/P), with risks of multifocal invasions, deep submucosal invasions, and submucosal fibrosis. Compared with piecemeal endoscopic membrane resection (EMR), endoscopic submucosal dissection (ESD) allows precise pathological evaluation after complete en bloc resection and lowers the recurrence rate.

Colonic ESD is technically difficult when the lesion is confounded with a thin colonic wall, colonic flexure, bowel motility, gravity direction, endoscope loop formation, poor endoscope maneuverability and fibrosis of the submucosal space [3–8]. Clear visualization of the submucosal layer is the key point of performing colonic ESD quickly and safely [9]. The classic way of maintaining visualization is to apply a transparent plastic cap to the endoscope tip and control the gravity direction by adjusting the body position [10, 11]. Novel methods such as saline-pocket ESD, pocket-creation ESD, dual-channel endoscope ESD, magnetic traction ESD, S–O clip-assisted ESD, cold snare-assisted ESD, robotic-assisted ESD, double-balloon platform-assisted ESD, etc. can also contribute to the visualization of the submucosal layer, although each method has its advantages and disadvantages [12–19].

Dual-clip and rubber band-assisted ESD (DCRB-ESD) is also an emerging method to achieve better traction during the ESD procedure. With clips and rubber band, the endoscopist can easily modify the traction direction against gravity without adjusting the body position. Recent studies have proven that this novel method is quicker and safer than conventional ESD (C-ESD) [7, 20, 21].

However, when confounded with the multiple factors mentioned above that can affect the performance of colonic ESD in clinical settings, whether DCRB-ESD will still perform better than C-ESD is not well estimated. In other words, data on DCRB-ESD on different clinical settings is limited. In our daily clinical practice, we also noticed that not all colonic ESD benefits from this novel technique. For some “easy” LST cases (small diameter, LST-G, satisfactory gravity direction, etc.), C-ESD might be quicker without introducing complication risks.

Therefore, we performed this retrospective case–control study to analyze the efficiency and safety of both DCRB-ESD and C-ESD in the management of colonic LSTs with varying technical difficulties in clinical settings.

## Methods

### Study design

This was a retrospective cohort study of patients who underwent colonic ESD for the treatment of LSTs at Binzhou Medical University Hospital, a regional medical center and general teaching hospital in Shandong Province of China. This study was approved by the ethical committee of Binzhou Medical University Hospital (Registration code 2022-LW-11). Informed consent was obtained from all patients before the procedure. All the data used in this study were collected through an endoscopic information system deployed in our medical center (Qingdao Medicon Co., Ltd, Qingdao, China). The STROBE checklist was followed to ensure the paper quality.

### Inclusion and exclusion criteria

All cases of colonic lesions referred for ESD treatment were included between Jan 1st, 2019 and Jan 1st, 2022, when the following inclusion criteria were met: (1) laterally spreading tumor (LST) ≥ 10 mm in size or adenomatous polyp with a broad base ≥ 10 mm and (2) no evidence of lymph node metastasis based on abdominal computed tomography (CT). Exclusion criteria included the following: (1) all rectal cases; (2) cases treated with hybrid ESD technique characterized by partial submucosal dissection followed by snare-assisted resection, due to its potential impact on the outcome of operation duration; (3) cases that were referred to C-ESD procedure but the trimming process was jammed for a long time, leading to unplanned application of DCRB-ESD (unplanned DCRB-ESD cases), due to the impact on the outcome of operation duration and the introduced heterogeneity.

### The semiquantitative model for the prediction of colorectal ESD difficulty

Yun-Shi Zhong et al. proposed a clinical score model for grading the technical difficulty of colorectal ESD (CS-CRESD), which predicts the probability of accomplishing colorectal ESD within 60 min and can be applied to grade the technical difficulty before the procedure [22]. This prediction model is based on the following four independent risk factors: tumor size (1 point when size 30–50 mm, 2 points when size ≥ 50 mm), the circumference of the lesion (2 points when circumference ≥ 2/3 and 0 points when less), lesion location (1 point for cecum, 2 points for flexure, 1 point for dentate line), and LST-NG lesion (1 point). The total score classifies the technical difficulty into easy (score = 0), intermediate (score = 1), difficult (score = 2–3), and very difficult (score ≥ 4).

### Decision of C-ESD or DCRB-ESD

Based on personally experience, the endoscopist (Q. Niu) evaluated the difficulty of the ESD procedure. The decision whether to perform C-ESD or DCRB-ESD was made by the endoscopist personally right before the markers were proposed.

### C-ESD and DCRB-ESD

ESD cases were all performed by an endoscopist (Q. Niu) who performed more than 100 gastrointestinal tract ESDs annually. The complication rate of ESDs performed by this endoscopist last year (2021) is 0.85% (two post-ESD gastral bleedings out of 236 gastrointestinal ESD cases, without any perforation).

The C-ESD procedure was conducted with a colonoscope (CF-HQ290I/CF-Q260JI, Olympus Co., Tokyo, Japan) and a high-frequency surgical device (VIO 200D, ERBE, Tübingen, Germany). A transparent plastic cap (D-201-11804; Olympus, Japan) was initially attached to the tip of the endoscope during this procedure to obtain better vision and maintain tension. Markers were pointed at least 5 mm to the edge of the LSTs using DualKnife (KD-650Q; Olympus, Tokyo, Japan). A mixed solution of indigo carmine, epinephrine, and saline was injected to fully lift the colonic mucosa layer with an injection needle (NM-200U-0523; Olympus, Japan). Then, electric trimming was conducted with a margin of 5 mm from the markers. Then, the trimming process was performed from the distal edge with the plastic cap introduced into the space of the submucosal layer. After the LSTs was fully resected, hemostatic forceps (HBF-16/1800; Micro-Tech Nanjing Co., Ltd, China) were used to perform coagulation to reduce bleeding risk. During this procedure, the patient’s position was adjusted when needed to control the direction of traction.

**Fig. 1:**
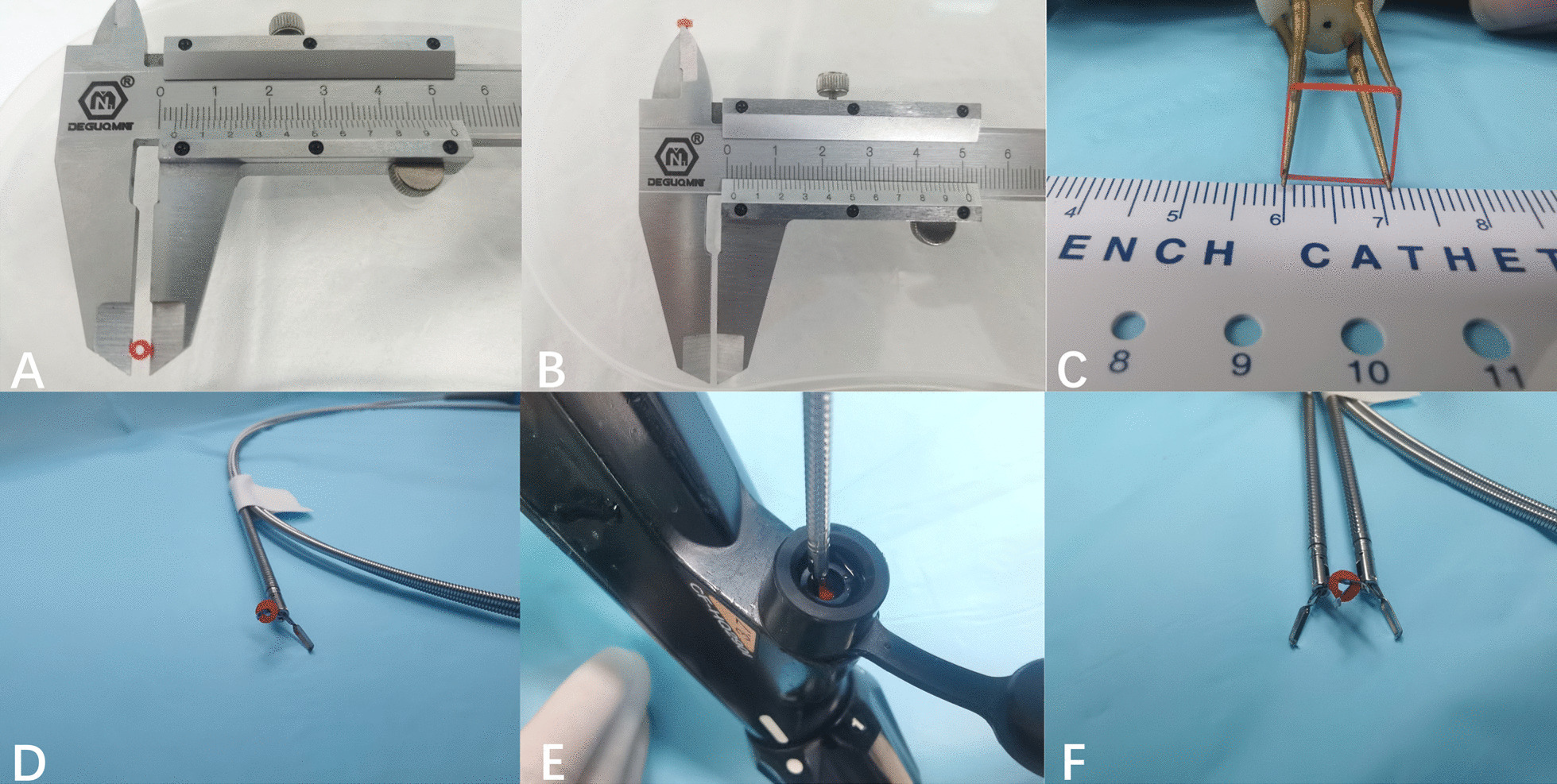
Composition of the traction device. **a** The outer diameter of the rubber band; **b** the inner diameter of a rubber band; **c** external rubber band elasticity; **d** the first clip and rubber band; **e** clamping the rubber band and entering the instrument channel; **f** a set with a dual-clip and rubber band externally

**Fig. 2:**
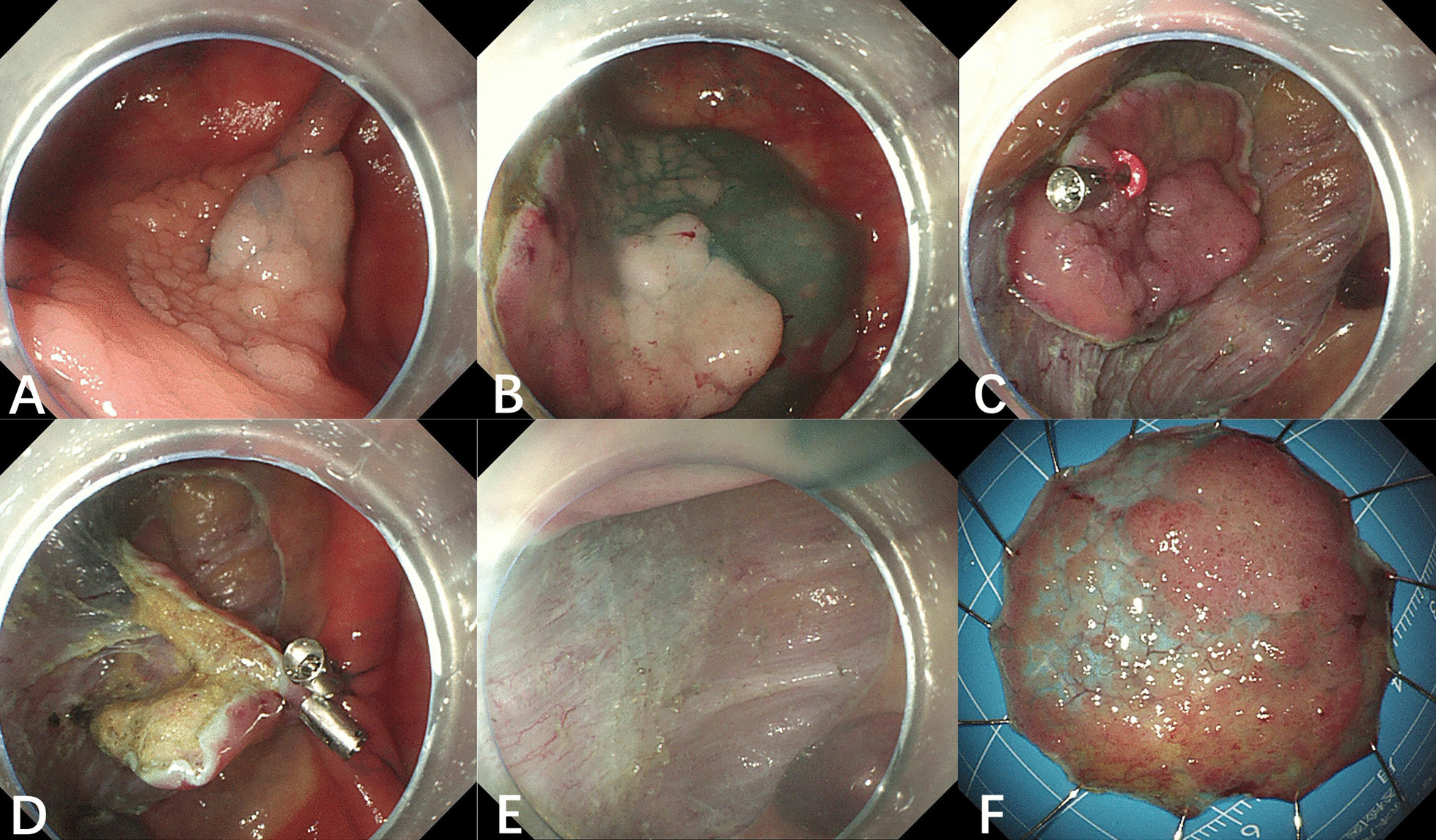
DCRB-ESD procedure. **a** LST-G(m) at the cecum; **b** after submucosal injection, circumferential incision and deep trimming were performed; **c** first clip with a rubber band attached on the edge of the LST; **d** fixation of the second clip grasping rubber band on the opposite wall to fully expose the submucosa; **e** dissection with traction; **f** specimen stretched on plate

The DCRB-ESD procedure was basically the same as C-ESD, with the extra help of a dual-clip and rubber band to obtain better vision and tension. Rubber bait bands (SeaKnight, Shanghai, China) were adopted. The band was approximately 3 mm in outer diameter, 1.5 mm in inner diameter, and 1 mm in thickness and could be directly deployed through the instrument channel of the endoscope (Fig. 1). When traction was needed during the ESD procedure, a clip (ROCC-D-26-195, Micro-Tech Nanjing Co., Ltd, China) holding the band edge was delivered through the instrument channel and was fixed on the margin of LSTs. Then, another clip was delivered to the colon. With one branch of the clip across the band, the clip was then dragged and fixed to the proper position of the colonic wall to gain traction. After resection, the clip fixed onto the colonic wall was removed by foreign body forceps (Fig. 2).

### Histopathological assessment

Specimens were fixed onto a foam plate and submerged into formalin after ESD according to the JSCCR guidelines criteria [2].

### Definitions

According to the JSCCR guidelines criteria, en bloc resection was defined as resection without fragmentation. Histologically complete resection (R0) was defined as en bloc resection with negative horizontal margin invasion (HM0) and negative vertical margin invasion (VM0). Curative resection was defined as R0 resection with no risk of lymph node metastasis. Perforation was defined as a complete hole through the colonic muscle during the treatment or clinical evidence upon postoperative radiological findings. Bleeding was defined as clinical evidence of bleeding after ESD.

The ESD procedure duration was defined as the time duration from submucosal injection to specimen retrieval.

### Outcomes

Operation duration was included as the primary outcome. The R0 resection rate, curative resection rate, tumor differentiation, tumor infiltration, bleeding, perforation, and additional surgery were included as secondary outcomes.

### Follow-up

All patients in this study were referred to a routine endoscopic follow-up procedure in the outpatient department.

### Statistical analysis and propensity score matching (PSM)

All calculations were conducted using the SPSS statistical software package (SPSS 26; Chicago, IL, USA). Continuous variables are presented as the mean and standard deviation. A chi-squared test was used for comparisons between categorical variables, and an independent t test was used for comparisons between continuous variables. Fisher’s exact test was used when necessary. Nonparametric statistics were performed when data did not match the normal distribution, where variables are presented as median and interquartile range. Two-sided P values less than 0.05 were considered statistically significant.

To reduce the heterogeneity of baselines, the propensity score matching (PSM) module of SPSS was employed. Variables employed in the PSM procedure were age, sex, LST size, circumference, LST location, LST classification and LST located in unfavorable location, with a matching ratio of 1:1. The match tolerance was set at 0.02.

## Results

### Outcomes before PSM

**Fig. 3:**
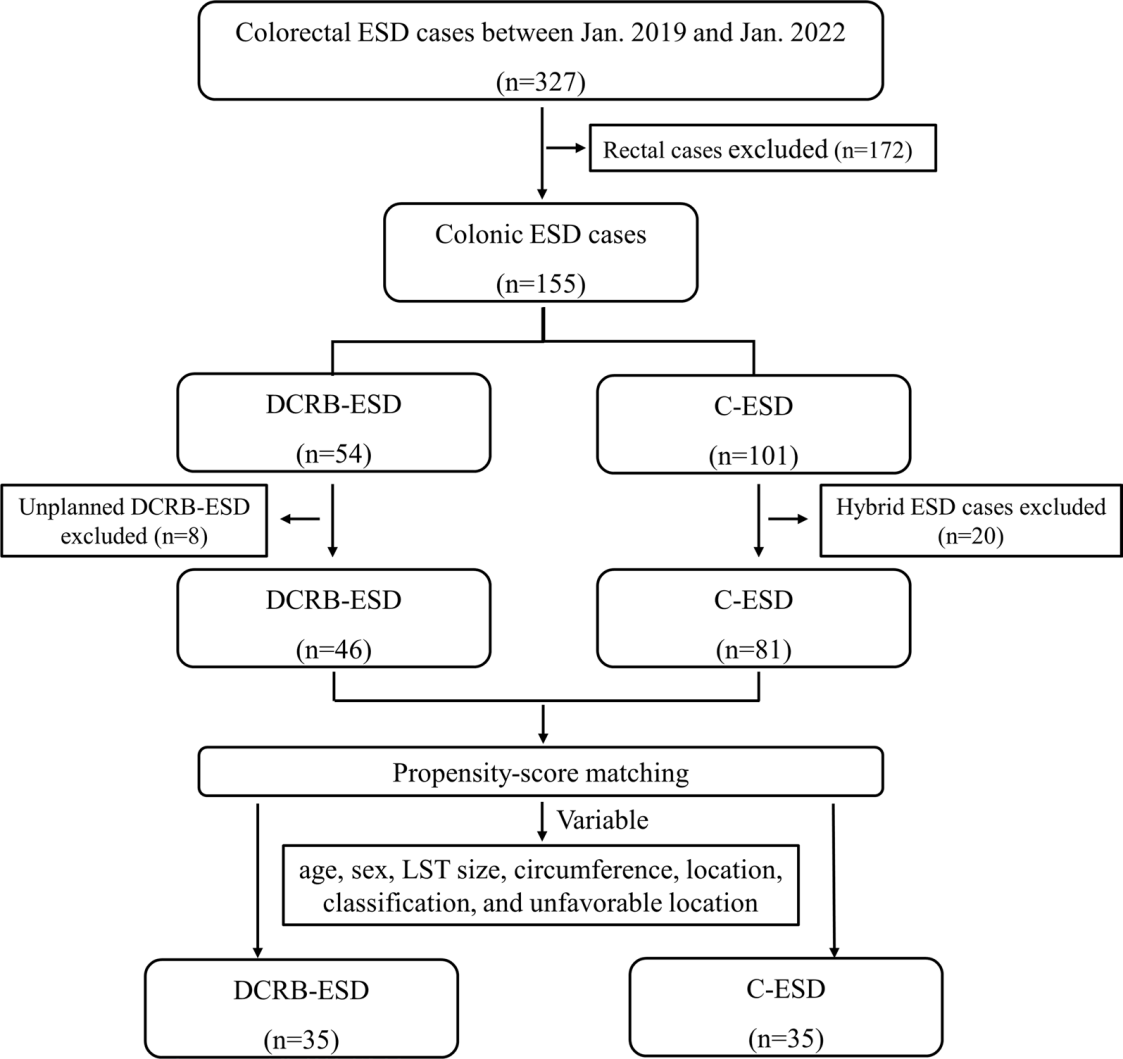
Flowchart depicting the patient selection process

Between Jan 1st 2019 and Jan 1st 2022, a total of 127 patients were enrolled, of whom 46 and 81 patients were grouped into the DCRB-ESD and C-ESD groups, respectively (Fig. 3). The baseline characteristics were well balanced between the two groups except for sex and LST classification (Table 1).

**Table 1:** Patient baseline characteristics before and after propensity score matching

Variables	Overall cohort			Matched cohort		
	C-ESD group (n = 81)	DCRB-ESD group (n = 46)	*p* value	C-ESD group (n = 35)	DCRB-ESD group (n = 35)	*p* value
Age	60.33 ± 12.60	60.65 ± 12.82	0.89	58.09 ± 18.09	59.46 ± 12.56	0.63
*Sex (male/female)*			0.00			1.0
Male	50	41		30	30	
Female	31	5		5	5	
*Location of lesions*			0.07			0.80
Rectal sigmoid junction	6	1		2	1	
Sigmoid colon	23	3		4	3	
Descending-sigmoid junction	7	4		1	4	
Descending colon	6	6		4	6	
Splenic flexure	1	2		1	1	
Transverse colon	11	6		8	5	
Hepatic flexure	6	6		3	2	
Ascending colon	14	9		7	5	
Cecum	7	9		5	8	
Unfavorable location	36	24	0.30	17	17	0.78
*Lesion size*			0.98			0.97
< 30 mm	55	32		22	23	
30–50 mm	24	13		12	11	
> 50 mm	2	1		1	1	
Lesion area (mm^2^) [Median, IQR]	500, 490.0	420, 480.0	0.63	500,675	360,525	0.30
*Lesion circumference ratio*			0.92			1.0
< 2/3	79	45		34	34	
> 2/3	2	1		1	1	
*LST classification*			0.00			0.81
LST-G	62	19		19	18	
LST-NG	19	27		16	17	
*Cases of each CS-CRESD score*			0.14			0.99
0	22	3		3	3	
1	23	18		14	15	
2	23	15		12	12	
3	10	7		4	3	
4	2	2		1	1	
5	1	1		1	1	

The median ESD procedure times were 32.0 and 41.0 min in the DCRB-ESD group and C-ESD group, respectively (*p* < 0.05). The operation durations of cases with different CS-CRESD scores were different (*p* < 0.05). In subgroups with a score of 0, DCRB-ESD spent more time than C-ESD (45.0 vs. 22.50, *p* < 0.05), but in subgroups with a score of 1 ~ 3, DCRB-ESD was faster than C-ESD (22.0 vs. 38.0, 42.0 vs. 52.0, 35.0 vs. 60.0, *p* < 0.05). In subgroups with a score of 4–5, the between-group operation duration was not significantly different due to limited cases, although DCRB-ESD was faster than C-ESD greatly (55.5 vs. 100.5 and *p* = 0.33, 146 vs. 172 and *p* = 1.0, respectively) (Table 2).

**Table 2:** ESD and postoperative outcomes before and after propensity score matching

Variables	Overall cohort			Matched cohort		
	C-ESD group (n = 81)	DCRB-ESD group (n = 46)	*p* value	C-ESD group (n = 35)	DCRB-ESD group (n = 35)	*p* value
Operation time of each CS-CRESD score subgroup (min)*						
0 [Median, IQR]	22.50, 14	45.0, –	0.05	31.00, –	45.00, –	0.3
1 [Median, IQR]	38.0, 11	22.0, 11	0.00	35.50,18	20.00,10	0.00
2 [Median, IQR]	52.0, 21	42.0, 18	0.00	51.50,22	43.00,19	0.04
3 [Median, IQR]	60.0, 27	35.0, 6	0.01	64.00,30	35.00, –	0.07
4 [Median, IQR]	100.5, –	55.5, –	0.33	128, –	58, –	0.32
5 [Median, IQR]	172, –	146, –	1.00	172, –	146, –	.032
*Post-ESD pathological diagnosis*			0.68			0.77
Sessile serrated adenoma	8	7		3	7	
Low-grade dysplasia adenoma	45	24		20	15	
High-grade dysplasia adenoma	19	8		7	7	
Mucosal cancer	3	2		2	2	
SM1 < 1000 μm	0	1		1	1	
SM2 > 1000 μm or deeper	6	4		2	3	
*En* bloc resection rate, n (%)	81/81 (100%)	46/46 (100%)	–	35/35	35/35	–
R0 resection rate, n (%)	75/81(92.59%)	45/46(97.83%)	0.10	33/35(94.3%)	34/35(97.1%)	1.0
Curative resection rate, n (%)	74/81(91.36%)	42/46(91.30%)	0.19	32/35(91.4%)	31/35(88.6%)	1.0
*Complications*						
Post-ESD bleeding	1/81 (1.23%)	1/46 (2.17%)	0.68	1(2.86%)	1(2.86%)	1.0
Perforation	0	0	–	0	0	–
Clip drop	–	1	–	0	1	–
Rubber band fracture	–	0		–	0	–
*Sets of DCRB used in each case*						
1	–	42		–	31	–
2	–	3		–	3	–
3	–	1		–	1	–
Additional surgery	3/81 (3.7%)	2/46(4.35%)	0.60	2/35(5.7%)	1/35(2.86%)	-

*The operation times for each subgroup did not conform to a normal distribution and are expressed as median and interquartile range (IQR). IQRs for subgroups of 4 and 5 were left blank due to lack of sufficient cases to calculate them

The en bloc resection rate in both groups was 100.0%. The R0 resection rates in both groups were not significantly different (92.59% vs. 97.83% in the C-ESD group and DCRB-ESD group, respectively, *p* = 0.10). The curative resection rates in both groups were not significantly different (91.36% in the C-ESD group and 91.30% in the DCRB-ESD group, *p* = 0.19). The post-ESD bleeding rates were 1.23% and 2.17%, respectively (*p* = 0.68). There were no perforation cases in either group. No adverse events, such as a clip dropping or rubber band rupturing, occurred in this study (Table 2).

When referring to lesion differentiation and depth of tumor invasion between the two groups, no significant difference was found (*p* = 0.68). Two patients in the DCRB-ESD group and three patients in the C-ESD group underwent additional surgery, and no metastasis was found (Table 2).

91.3% of the DCRB-ESDs were performed with one set of DCRB. Two sets of DCRBs were used separately in three lesions due to large lesion area, and three sets were used in one LST-NG lesion due to unclear visualization of the submucosa.

### Outcomes after PSM

**Fig. 4:**
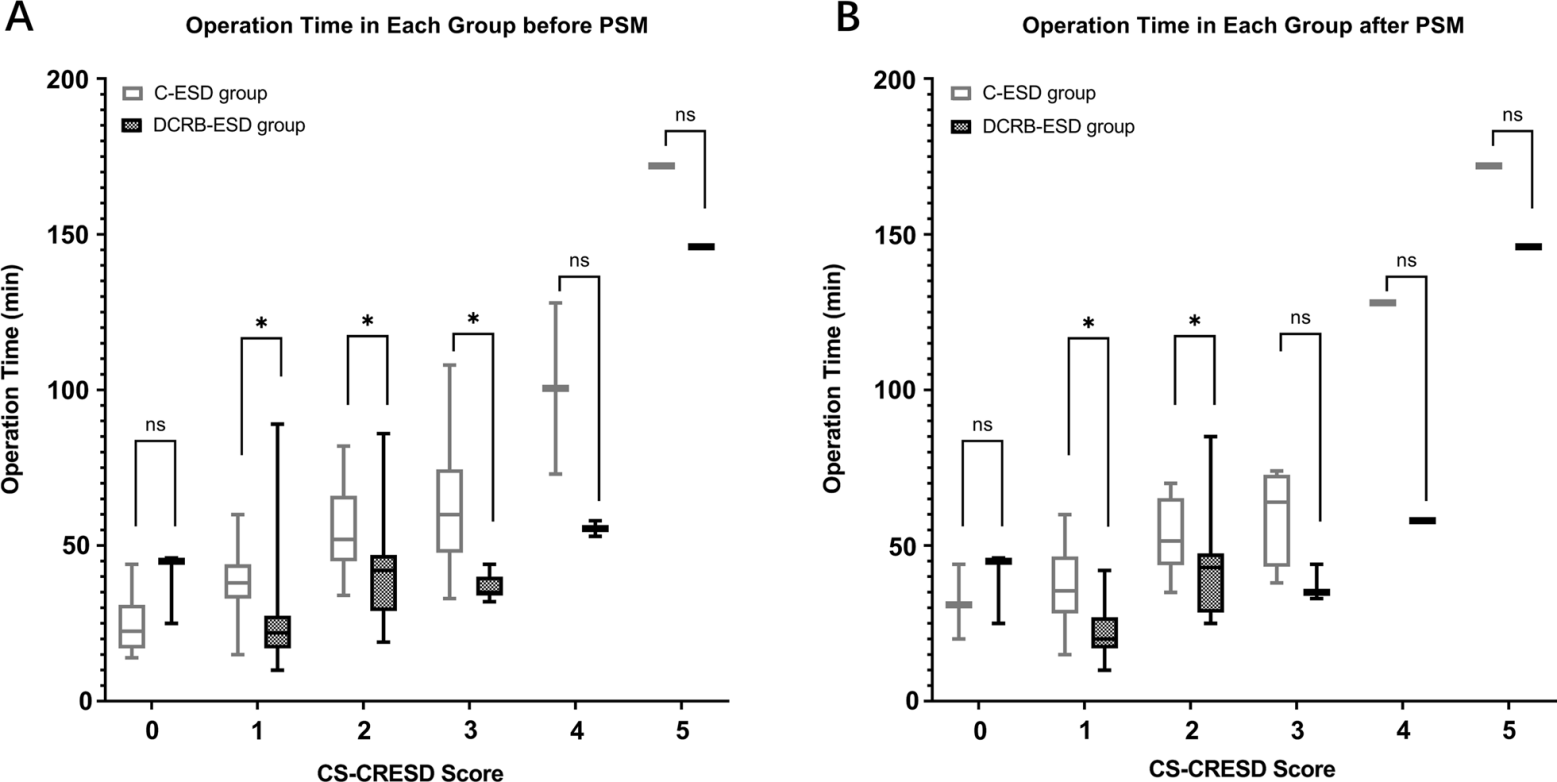
The operation time of DCRB-ESD and C-ESD based on CS-CRESD before and after PSM. **a** Before the PSM, **b** after the PSM. *, statistically significant. ns, not statistically significant

Since LST classification could potentially influence the ESD procedure time, PSM was performed, and the unbalance was corrected. The results were basically the same. Although the statistical significances in some subgroups were influenced, the overall results still follow the same trend after PSM (Fig. 4).

## Discussion

Our study proved the value of DCRB in the management of colonic LST cases. This technique generally shortens the operation duration and it contributes less when facing easy colonic LST cases.

Our data proved that the DCRB could improve ESD efficiency. Overall, the operation duration with DCRB is significantly reduced. DCRB-ESD could generally shorten the operation duration, especially for difficult LSTs. The operation times of cases with CS-CRESD of 1–3 were shorter, when DCRBs were used. Moreover, the interquartile range of operation duration in the DCRB-ESD group was smaller than that of C-ESD, indicating that the application of DCRB can maintain the stability of the ESD technique in cases of 1–3 points of CS-CRESD (Fig. 4). Due to the insufficient number of cases in subgroups with CS-CRESD of 4 or 5, the operation duration was not significantly different. However, since DCRB showed stable performance in subgroups with lower scores, we believe that further study with more cases would show the value of DCRB in cases with higher difficulty scores.

In this study, DCRB-ESD showed no superiority over C-ESD in terms of operation duration in cases with a CS-CRESD score of 0 before PSM. After PSM, the median operation time of DCRB-ESD was still larger than that of C-ESD, although the difference was not statistically significant. This insignificant outcome after PSM is mainly attributed to the limited number of cases with a score of 0. When planning ESD treatment, the endoscopist tends to not apply DCRB in easy cases. The reason is it spends about 3–5 min to place DCRB(s) and therefore the overall operation time is affected. If the endoscopist evaluates that the extra time spent on DCRB(s) cannot offset the saved operation time, DCRB-ESD will not be chosen. In other words, we think that whether DCRB-ESD shortens operation time of easy LST cases depends largely on the experience of the endoscopist. Since the endoscopist (Q. Niu) in this study is experienced in ESD procedure, most of the easy cases were referred to C-ESD. The results proved that fine technique in C-ESD can offset the time saved in DCB-ESD in easy cases.

Our data also proved that DCRB-ESD was safe. As proven by many researchers, the DCRB-ESD technique can reduce postoperative complications and increase the en bloc resection rate, R0 resection rate, and curative resection rate [7, 21]. In our study, there was no perforation or infection related to DCRB-ESD. Only one patient suffered post-ESD bleeding in the DCRB-ESD group (1/46, 2.17%). This post-ESD bleeding lesion was located at the rectosigmoid junction. The bleeding site was located within the post-ESD ulcer, with no evidence of bleeding in the opposite wall, which was the site to place the second clip. The data in this study showed no significant difference referring to the above parameters, which could attribute to the highly experience of the endoscopist.

In our study, fishing bait bands were adopted (Fig. 1). Compared with other commercial sets, these rubber bands were easy to obtain and inexpensive. No infections or rupture of rubber bands occurred during the study, and the traction of the mucous membrane was continuous and strong. Combined with the adjustment of air volume, they can provide stable traction during ESD. During the ESD procedure, this kind of bait rubber bands can be easily transmitted through the instrument channel, and the clips on the colon wall can be removed or repositioned with the help of snare or foreign body forceps. During the study, the damage of the colonic wall or specimens by DCRB was not observed. Although one clip dropped from the specimen during the operation, it did not cause serious consequences.

The placement procedure of DCRB is to first identify the direction of gravity and then judge the operability range of the endoscope to find a proper clip placement position. Before circumcision or semi circumcision, a slightly larger anal margin of LSTs should be reserved to avoid tissue damage by the first clip. When placing the DCRB, it is necessary to avoid drawing it into the deep part of the cutting margin. We also tried to use multiple sets of DCRBs when facing difficult lesions, and all achieved good traction.

In this study, eight unplanned DCRB-ESD cases were excluded from the analysis because time spent on the unplanned jammed trimming process can introduce heterogeneity if these cases were allocated to DCRB-ESD group. The first and second cases were Niu's early attempts of DCRB-ESD, and they both scored 2 points on CS-CRESD score. The overall operation duration was 39 min and 93 min separately, and the time after the application of DCRB was 5 min and 13 min. The next 6 cases scored 0 ~ 3 points were performed by trainee operators initially and were jammed during the trimming process for a long time during the operation. After the application of DCRB, visualization was improved. These ESDs were successfully completed with no complications. After applying DCRB, these 6 unplanned cases spent 18.25 ± 14.53 min to finish the ESD procedure. Although these unplanned DCRB-ESD cases were not included in the analysis, these data proved that DCRB could improve work efficiency in both trainees and experts, as well as the irreplaceable role of DCRB-ESD in difficult cases (Table 3).

**Table 3:** Characteristics of unplanned DCRB-ESD cases

	Age	Gender	Location	Size (mm)	circumference	Morphology	CS-CRESD score	ESD total operation time(min)	After using DCRB time (min)	Operator
Case 1	70	Female	Cecum	30–50	< 2/3	LST-G	2	39	5	Niu
Case 2	40	Male	flexure	< 30	< 2/3	LST-G	2	93	13	Niu
Case 3	61	Male	flexure	< 30	< 2/3	LST-NG	3	40	23	Niu assisted
Case 4	81	Male	Transverse colon	< 30	< 2/3	LST-G	0	71	21	Niu assisted
Case 5	66	Male	Cecum	< 30	< 2/3	LST-G	1	106	6	Niu assisted
Case 6	68	Male	Cecum	> 50	< 2/3	LST-G	3	93	33	Niu assisted
Case 7	69	Male	Descending colon	< 30	< 2/3	LST-G	0	75	43	Niu assisted
Case 8	64	Female	Ascending colon	< 30	< 2/3	LST-G	0	63	2	Niu assisted

In our study, PSM was introduced to manage the heterogeneity [23]. We should admit this method could also introduce limitations [24]. Other limitations of this study include its retrospective design, an insufficient number of cases, and no comparison with other traction methods, calling for more data of prospective studies.

## Conclusion

DCRB-ESD is valuable in the management of LSTs of high technical difficulty in the colon. We recommend CS-CRESD to grade the technical difficulty before the ESD procedure. For intermediate and difficult LSTs, DCRB-ESD is an efficient and safe method. For easy cases, the ability of DCRB-ESD to reduce the operation duration depends largely on the experience of the endoscopist.

## Data Availability

The datasets used and/or analyzed during the current study are available from the corresponding author on reasonable request.

## Abbreviations

LST: Lateral spreading tumors
EMR: Endoscopic membrane resection
ESD: Endoscopic submucosal dissection
DCRB-ESD: Dual-clip and rubber band-assisted ESD
C-ESD: Conventional ESD
PSM: Propensity score matching
JSGE: Japan Gastroenterological Endoscopy Society
LST-G: Granular type of LST
LST-NG: Nongranular type of LST
SSA/P: Adenocarcinomas or sessile serrated adenomas/polyps
CS-CRESD: Clinical score model for grading the technical difficulty of colorectal ESD
R0: Complete resection
HM0: Negative horizontal margin invasion
VM0: Negative vertical margin invasion

## References

[1] The Paris endoscopic classification of superficial neoplastic lesions: esophagus, stomach, and colon: November 30 to December 1, 2002. Gastrointest Endosc. 2003;58(6 Suppl):S3–43. 10.1016/s0016-5107(03)02159-x 14652541

[2] Hashiguchi Y, Muro K, Saito Y, Ito Y, Ajioka Y, Hamaguchi T, Hasegawa K, Hotta K, Ishida H, Ishiguro M (2020). Japanese Society for Cancer of the Colon and Rectum (JSCCR) guidelines 2019 for the treatment of colorectal cancer. Int J Clin Oncol.

[3] Zhang X, Ly EK, Nithyanand S, Modayil RJ, Khodorskiy DO, Neppala S, Bhumi S, DeMaria M, Widmer JL, Friedel DM (2020). Learning curve for endoscopic submucosal dissection with an untutored, prevalence-based approach in the United States. Clin Gastroenterol Hepatol.

[4] Boda K, Oka S, Tanaka S, Nagata S, Kunihiro M, Kuwai T, Hiraga Y, Furudoi A, Nakadoi K, Okanobu H (2020). Real-world learning curve analysis of colorectal endoscopic submucosal dissection: a large multicenter study. Surg Endosc.

[5] Fukunaga S, Nagami Y, Shiba M, Sakai T, Maruyama H, Ominami M, Otani K, Hosomi S, Tanaka F, Taira K (2019). Impact of preoperative biopsy sampling on severe submucosal fibrosis on endoscopic submucosal dissection for colorectal laterally spreading tumors: a propensity score analysis. Gastrointest Endosc.

[6] Saunders BP, Tsiamoulos ZP (2016). Endoscopic mucosal resection and endoscopic submucosal dissection of large colonic polyps. Nat Rev Gastroenterol Hepatol.

[7] Jacques J, Charissoux A, Bordillon P, Legros R, Rivory J, Hervieu V, Albouys J, Guyot A, Ponchon T, Sautereau D (2019). High proficiency of colonic endoscopic submucosal dissection in Europe thanks to countertraction strategy using a double clip and rubber band. Endosc Int Open.

[8] Fung TLD, Chow CWS, Chan PT, Kwok KH (2020). Review on colorectal endoscopic submucosal dissection focusing on the technical aspect. Surg Endosc.

[9] Abe S, Wu SYS, Ego M, Takamaru H, Sekiguchi M, Yamada M, Nonaka S, Sakamoto T, Suzuki H, Yoshinaga S (2020). Efficacy of current traction techniques for endoscopic submucosal dissection. Gut Liver.

[10] Ko WJ, Song GW, Hong SP, Kwon C-I, Hahm KB, Cho JY (2016). Novel 3D-printing technique for caps to enable tailored therapeutic endoscopy. Dig Endosc.

[11] Inoue H, Endo M, Takeshita K, Yoshino K, Muraoka Y, Yoneshima H (1992). A new simplified technique of endoscopic esophageal mucosal resection using a cap-fitted panendoscope (EMRC). Surg Endosc.

[12] Harada H, Nakahara R, Murakami D, Suehiro S, Ujihara T, Sagami R, Katsuyama Y, Hayasaka K, Amano Y (2019). Saline-pocket endoscopic submucosal dissection for superficial colorectal neoplasms: a randomized controlled trial (with video). Gastrointest Endosc.

[13] Takezawa T, Hayashi Y, Shinozaki S, Sagara Y, Okada M, Kobayashi Y, Sakamoto H, Miura Y, Sunada K, Lefor AK (2019). The pocket-creation method facilitates colonic endoscopic submucosal dissection (with video). Gastrointest Endosc.

[14] Sakamoto N, Osada T, Shibuya T, Beppu K, Matsumoto K, Mori H, Kawabe M, Nagahara A, Otaka M, Ogihara T (2009). Endoscopic submucosal dissection of large colorectal tumors by using a novel spring-action S-O clip for traction (with video). Gastrointest Endosc.

[15] Dobashi A, Storm AC, Wong Kee Song LM, Deters JL, Miller CA, Tholen CJ, Gostout CJ, Rajan E (2019). An internal magnet traction device reduces procedure time for endoscopic submucosal dissection by expert and non-expert endoscopists: ex vivo study in a porcine colorectal model (with video). Surg Endosc.

[16] Zhang Q, Xing T-Y, Wang Z (2019). A snare combined with endoclips to assist in endoscopic submucosal dissection for intraepithelial neoplasia in the entire colon and rectum. Scand J Gastroenterol.

[17] Turiani Hourneaux de Moura D, Aihara H, Jirapinyo P, Farias G, Hathorn KE, Bazarbashi A, Sachdev A, Thompson CC (2019). Robot-assisted endoscopic submucosal dissection versus conventional ESD for colorectal lesions: outcomes of a randomized pilot study in endoscopists without prior ESD experience (with video). Gastrointest Endosc.

[18] Jacques J, Albouys J, Guyot A, Geyl S, Legros R, Chaput U, Pioche M (2019). Endoscopic submucosal dissection of a laterally spreading tumor in the right colon with a gastroscope after shortening the colon using a new double-balloon platform. Endoscopy.

[19] Yoshida M, Takizawa K, Suzuki S, Koike Y, Nonaka S, Yamasaki Y, Minagawa T, Sato C, Takeuchi C, Watanabe K (2018). Conventional versus traction-assisted endoscopic submucosal dissection for gastric neoplasms: a multicenter, randomized controlled trial (with video). Gastrointest Endosc.

[20] Faller J, Jacques J, Oung B, Legros R, Rivory J, Subtil F, Saurin J-C, Robinson P, Ponchon T, Pioche M (2020). Endoscopic submucosal dissection with double clip and rubber band traction for residual or locally recurrent colonic lesions after previous endoscopic mucosal resection. Endoscopy.

[21] Bordillon P, Pioche M, Wallenhorst T, Rivory J, Legros R, Albouys J, Lepetit H, Rostain F, Dahan M, Ponchon T (2021). Double-clip traction for colonic endoscopic submucosal dissection: a multicenter study of 599 consecutive cases (with video). Gastrointest Endosc.

[22] Li B, Shi Q, Xu E-P, Yao L-Q, Cai S-L, Qi Z-P, Sun D, He D-L, Yalikong A, Lv Z-T (2021). Prediction of technically difficult endoscopic submucosal dissection for large superficial colorectal tumors: a novel clinical score model. Gastrointest Endosc.

[23] Haukoos JS, Lewis RJ (2015). The propensity score. JAMA.

[24] Dong J, Zhang JL, Zeng S, Li F (2020). Subgroup balancing propensity score. Stat Methods Med Res.

